# Real‐World nivolumab dosing patterns and safety outcomes in patients receiving adjuvant therapy for melanoma

**DOI:** 10.1002/cam4.5061

**Published:** 2022-07-25

**Authors:** Wolfram Samlowski, Nicholas J. Robert, Liwei Chen, Brad Schenkel, Catherine Davis, Andriy Moshyk, Srividya Kotapati, Tayla Poretta, Jeffrey S. Weber

**Affiliations:** ^1^ Comprehensive Cancer Centers of Nevada and University of Nevada Las Vegas Kirkorian School of Medicine Las Vegas Nevada USA; ^2^ University of Nevada School of Medicine Reno Nevada USA; ^3^ McKesson Life Sciences The Woodlands Texas USA; ^4^ Bristol Myers Squibb Princeton New Jersey USA; ^5^ Perlmutter Cancer Center NYU School of Medicine New York New York USA

**Keywords:** adjuvant therapy, dosing interval, flat dosing, melanoma, nivolumab, real‐world, safety, tolerability

## Abstract

**Background:**

Nivolumab at a dose of 480 mg every 4 weeks (Q4W) is approved for the adjuvant treatment of melanoma. However, real‐world data on this regimen are limited in this setting.

**Methods:**

This retrospective observational study utilized data from the US Oncology Network iKnowMed electronic health record database and patient medical charts. Eligible patients were diagnosed with melanoma and received adjuvant nivolumab monotherapy from March to August 2018. Patients were grouped by dosing regimen: cohort 1 (C1), de novo nivolumab 480 mg Q4W; cohort 2 (C2), switched to nivolumab 480 mg Q4W after nivolumab 240 mg or 3 mg/kg every 2 weeks (Q2W); cohort 3 (C3), de novo nivolumab 3 mg/kg Q2W; or cohort 4 (C4), de novo nivolumab 240 mg Q2W. Patients were followed for up to 12 months. Duration of therapy and safety/tolerability were assessed.

**Results:**

One hundred ninety‐one patients were included (C1, *n* = 40; C2, *n* = 74; C3, *n* = 22; C4, *n* = 55). Duration of therapy was relatively consistent across cohorts (median, 10.3 months; range, 8.3–10.7). Likewise, proportions of patients experiencing treatment‐related adverse events (TRAEs) were similar (range, 54.5%–60.1%), as were the most common events (fatigue, rash, diarrhea, hypothyroidism, nausea, and pruritus). However, proportions experiencing ‘significant’ TRAEs varied between cohorts. Proportions discontinuing treatment were relatively consistent across cohorts. Propensity score matching and sensitivity analyses generally supported the unadjusted findings.

**Conclusions:**

Real‐world safety profiles of nivolumab 240 mg Q2W and 480 mg Q4W were similar, and both were comparable to the well‐documented safety of weight‐based dosing (3 mg/kg Q2W), further supporting their approval and use in the adjuvant setting for melanoma.

## Graphical abstract

Real‐word data on use of the flat‐dose nivolumab 480 mg Q4W regimen for the adjuvant treatment of melanoma are limited. Findings from the current study suggest that the real‐world safety and tolerability profiles of nivolumab 240 mg Q2W and 480 mg Q4W are similar and that both are comparable to weight‐based dosing (3 mg/kg Q2W), adding to existing data supporting their approval and use as adjuvant treatments for melanoma.

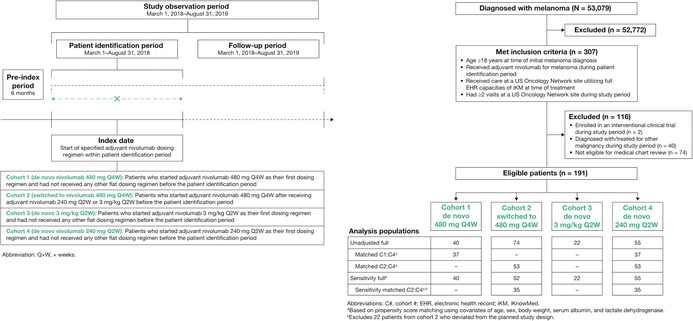

## 1 INTRODUCTION

On the basis of data from the CheckMate 238 trial, the programmed death‐1 (PD‐1) inhibitor nivolumab was approved in 2017 for the adjuvant treatment of patients with melanoma with involvement of lymph nodes or those with metastatic disease who had undergone complete surgical resection.^1^, ^2^ At the time of writing, nivolumab is approved for the adjuvant setting in several countries and regions, including the United States, Europe, and Japan.

Like many other monoclonal antibodies, nivolumab was historically dosed according to patient body weight because this was believed to decrease interpatient variability in drug exposure versus flat dosing.^3^, ^4^ However, clinical studies also indicated that nivolumab has linear pharmacokinetics and a wide therapeutic index,^5^, ^6^, ^7^, ^8^ suggesting the potential for flat dosing, regardless of the patient's weight. As an alternative to traditional weight‐based dosing regimens, flat dosing schedules offer several potential benefits to healthcare professionals, including shortened and simplified drug preparation, decreased likelihood of dosing errors, and reduced burden.

On the basis of the known wide therapeutic index for nivolumab, flat‐dose regimens were investigated with population pharmacokinetic modeling and exposure–response analyses, suggesting that efficacy and safety exposure–response relationships, clinical safety, and benefit/risk profiles for nivolumab 240 mg every 2 weeks (Q2W) and 480 mg every 4 weeks (Q4W) were similar to those for the original weight‐based 3 mg/kg Q2W regimen in patients with advanced cancers.^3^, ^4^, ^9^ Because of these observations, the 240 mg Q2W regimen, and more recently the 480 Q4W regimen, were approved by the US Food and Drug Administration and the European Medicines Agency for various cancer indications, including the adjuvant treatment of melanoma.^10^, ^11^ In addition to the aforementioned advantages of flat dosing over weight‐based dosing, the less frequent Q4W dosing schedule would provide time savings for patients.

Although some data on the adjuvant use of nivolumab 480 mg Q4W in patients with melanoma are now available from the phase 3 CheckMate 915 trial,^12^, ^13^ published data on this regimen in the adjuvant setting for melanoma remain limited, both from clinical trials and real‐world studies. To address the scarcity of real‐world data, the current study examined data from cohorts of patients receiving nivolumab 480 mg Q4W for the adjuvant treatment of melanoma in the US community oncology setting. To better contextualize patient outcomes, the study was designed to compare these data with those from real‐world cohorts receiving flat‐dose 240 mg Q2W and weight‐based 3 mg/kg Q2W regimens.

## 2 MATERIALS AND METHODS

### 2.1 Study design and participants

**FIGURE 1:**
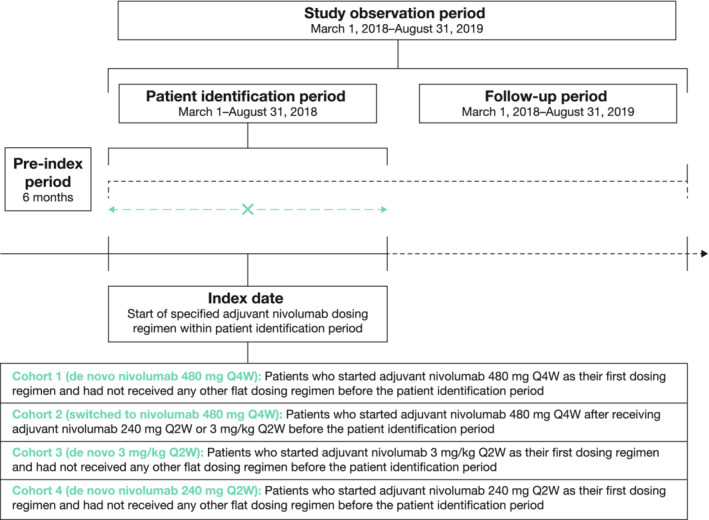
Study design and cohort allocation. Q × W indicates every × weeks.

This retrospective observational study utilized data from the US Oncology Network iKnowMed (iKM) electronic health record (EHR) database, supplemented by medical chart review. The study population comprised adult patients (≥18 years old) who were diagnosed with melanoma and received adjuvant nivolumab monotherapy for melanoma at a US Oncology Network site between March 1 and August 31, 2018 (the protocol‐defined *patient identification period*; Figure 1). Eligible patients must also have received care at a US Oncology Network site utilizing the full EHR capacities of the US Oncology Network iKM database at the time of treatment and have had ≥2 visits at a US Oncology Network site between March 1, 2018, and August 31, 2019 (the protocol‐defined *study observation period*; Figure 1). Exclusion criteria were diagnosis of and/or treatment for another malignancy and enrollment in a clinical trial. Patients who were ineligible for medical chart review were also excluded.

### 2.2 Cohort assignment and indexing

Identified eligible patients were assigned an analysis cohort based on their adjuvant nivolumab dosing within the patient identification period as shown in Figure 1. The drug index date represented the first date of adjuvant nivolumab dosing during the patient identification period for patients in cohorts 1, 3, and 4, or the first date of nivolumab 480 mg Q4W dosing during the patient identification period for patients in cohort 2. Determination of prior treatment with adjuvant nivolumab (for assignment to cohort 2) was based on assessment of data collected in the 6 months immediately before the drug index date (pre‐index period). All patients were followed from their index date for up to 12 months, to the end of the study observation period, to the date of last visit, or to the date of death, whichever occurred first. The last visit was defined as the final physical encounter, detected by vital signs records within the US Oncology Network iKM EHR database. For patients who started an adjuvant nivolumab regimen during the patient identification period, but then switched to another dosing regimen during follow‐up, data were analyzed up until the date of that switch.

### 2.3 Endpoints

The protocol‐defined prespecified primary endpoints were patient demographic and clinical characteristics, duration of therapy, safety, and reasons for treatment discontinuation. Patient demographic and clinical characteristics were based primarily on data from the US Oncology Network iKM EHR database, with additional information provided by medical chart review. Reported demographic and clinical characteristics were those documented on the closest date to the drug index date (±30 days). Exceptions were disease stage (recorded by physician at diagnosis or on closest date to diagnosis using the TNM classification system) and patient age (recorded on the exact index date). Duration of therapy was the time from drug index date to last administration date, including any treatment delays of no more than 180 consecutive days, as documented in the US Oncology Network iKM EHR database. Patients without a recorded second administration of adjuvant nivolumab were defined as having a treatment duration of 1 day. Safety was assessed by the incidence of treatment‐related adverse events (TRAEs) and ‘significant’ TRAEs. A TRAE was protocol‐defined as any adverse event that occurred after the initiation of adjuvant nivolumab for melanoma and that was attributed explicitly to adjuvant nivolumab as documented in a patient's medical chart. ‘Significant’ TRAEs were protocol‐defined as any TRAE that led to any of the following outcomes: withheld dose, altered dose and/or schedule, permanent treatment discontinuation, hospitalization, or emergency department visit. Safety was assessed from drug index date to end of follow‐up or date of death, whichever came first. For patients discontinuing therapy, TRAEs occurring up to 30 days after discontinuation were reported. Reasons for treatment discontinuation were determined by medical chart review.

### 2.4 Analyses

For demographic and baseline clinical characteristics, chi‐square testing (parametric) or the Fisher exact test (nonparametric) was used to assess differences between categorical variables when patient counts for individual categories were ≥5. Depending on normality, ANOVA/*t*‐tests or Kruskal–Wallis tests were conducted for continuous variables. Duration of therapy was estimated using Kaplan–Meier methodology.^14^ Safety was summarized using descriptive statistics with AEs defined per the National Cancer Institute's Common Terminology Criteria for Adverse Events (CTCAE) version 5. However, assignment of specific severity grading (grade 1–5) was not feasible in this study based on the abstracted information from patients' medical charts. ‘Significant’ TRAEs were instead protocol‐defined as described above. To control for potential differences between cohorts in patient demographic and baseline clinical characteristics and to minimize potential selection bias, propensity score matching was performed using covariates of age, sex, body weight, baseline serum albumin and lactate dehydrogenase. All statistical analyses were conducted using SAS 9.4 (SAS Institute Inc., Cary, NC).

## 3 RESULTS

### 3.1 Patient Populations

**FIGURE 2:**
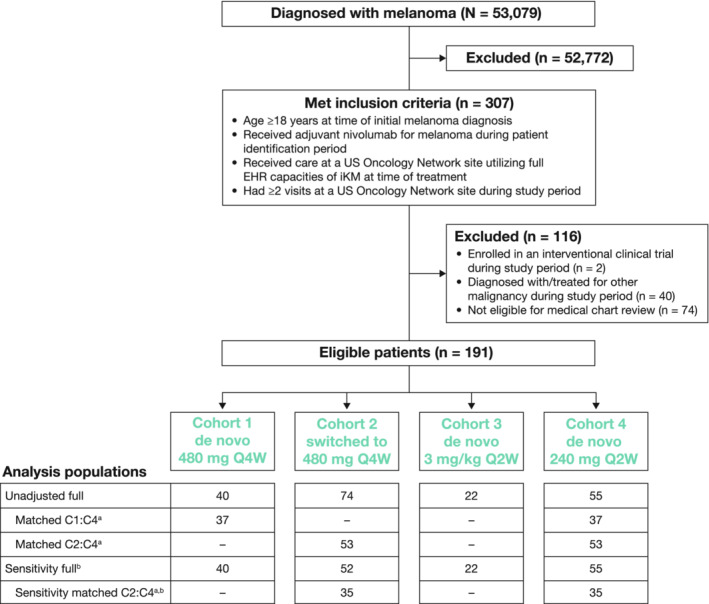
Patient disposition and cohort analysis populations. C# indicates cohort #; EHR, electronic health record; iKM, iKnowMed; Q × W, every × weeks. ^a^Based on propensity score matching using covariates of age, sex, body weight, serum albumin, and lactate dehydrogenase. ^b^Excludes 22 patients from cohort 2 who deviated from the planned study design.

Overall, 191 patients met the eligibility criteria and were assigned across the 4 cohorts (Figure 2). Subgroups of this unadjusted population were identified by propensity score matching to facilitate matched analyses for cohorts 1 versus 4 (C1:C4) and 2 versus 4 (C2:C4). A matched population was not defined for cohort 3 because of the small sample size for the unadjusted population (*n* = 22). Although 74 patients were initially assigned to cohort 2 (i.e., switched to adjuvant nivolumab 480 mg Q4W), 54 of these patients were found to have deviated from the planned study design as their drug index dates were recorded during their prior treatment with either 240 mg Q2W or 3 mg/kg Q2W (i.e., before the switch to 480 mg Q4W). Moreover, 22 of these 54 patients had switched to the 480 mg Q4W dosing regimen after the end of the patient identification period. No patients in the other cohorts experienced any deviations. Because including the 22 patients who switched to 480 mg Q4W after the study's predefined enrollment period could influence the observed outcomes, sensitivity analyses were performed after excluding these 22 patients (Figure 2).

### 3.2 Patient demographic and baseline clinical characteristics

In the unadjusted full population (*N* = 191), median age was 61 years, and most patients were male (60.7%), Caucasian (85.3%), and had stage III disease at initial diagnosis (81.7%). Most had an Eastern Cooperative Oncology Group performance score (ECOG PS) of 0–1 (75.4%) and a Charlson Comorbidity Index risk score of 0 (71.7%). Patient demographic and clinical characteristics were generally comparable across the 4 cohorts (Table 1). However, there was an observed variance for the ECOG PS categories, primarily driven by cohorts 1 and 2 having larger proportions of patients with missing ECOG data versus the other cohorts. In addition, there was a trend toward lower mean/median body weight for patients in cohort 3 versus the other cohorts.

**TABLE 1:** Demographic and clinical characteristics of patients in the unadjusted full population (*N* = 191)

Characteristic^a^	All patients (*N* = 191)	Cohort 1 (*N* = 40)	Cohort 2 (*N* = 74)	Cohort 3 (*N* = 22)	Cohort 4 (*N* = 55)
Median age (range) at index, years	61 (20–90)	65 (24–87)	60 (20–90)	59 (27–84)	61 (20–87)
Age group at index, *n* (%)					
<65 years	109 (57.1)	19 (47.5)	46 (62.2)	12 (54.5)	32 (58.2)
≥65 years	82 (42.9)	21 (52.5)	28 (37.8)	10 (45.5)	23 (41.8)
Race, *n* (%)					
Caucasian	163 (85.3)	37 (92.5)	65 (87.8)	16 (72.7)	45 (81.8)
Black or African American	1 (0.5)	0	1 (1.4)	0 (0.0)	0
Asian	1 (0.5)	0	0	1 (4.5)	0
American Indian or Alaska native	1 (0.5)	0	0	1 (4.5)	0
Other	25 (13.1)	3 (7.5)	8 (10.8)	4 (18.2)	10 (18.2)
Sex, *n* (%)					
Female	75 (39.3)	14 (35.0)	28 (37.8)	10 (45.5)	23 (41.8)
Male	116 (60.7)	26 (65.0)	46 (62.2)	12 (54.5)	32 (58.2)
Weight, kg					
Patients with available data	189	39	73	22	55
Median (range)	85.8 (30.6–153.9)	93.4 (62.1–143.3)	83.9 (40.5–153.9)	70.8 (30.6–139.7)	88.9 (43.1–143.8)
LDH group, *n* (%)^b^					
< ULN	168 (88.0)	38 (95.0)	62 (83.8)	21 (95.5)	47 (85.5)
Within normal limits	85 (44.5)	20 (50.0)	31 (41.9)	9 (40.9)	25 (45.5)
> ULN	1 (0.5)	0	1 (1.4)	0	0
Stage at diagnosis, *n* (%)					
II (unspecified)	1 (0.5)	1 (2.5)	0	0	0
IIA	5 (2.6)	0	2 (2.7)	0	3 (5.5)
IIB	5 (2.6)	3 (7.5)	2 (2.7)	0	0
IIC	4 (2.1)	3 (7.5)	0	0	1 (1.8)
III (unspecified)	24 (12.6)	1 (2.5)	12 (16.2)	4 (18.2)	7 (12.7)
IIIA	26 (13.6)	4 (10.0)	10 (13.5)	4 (18.2)	8 (14.5)
IIIB	60 (31.4)	15 (37.5)	23 (31.1)	6 (27.3)	16 (29.1)
IIIC	46 (24.1)	8 (20.0)	19 (25.7)	7 (31.8)	12 (21.8)
IV	13 (6.8)	5 (12.5)	2 (2.7)	0	6 (10.9)
Not documented	7 (3.7)	0	4 (5.4)	1 (4.5)	2 (3.6)
*BRAF* status, *n* (%)					
Wild type	43 (22.5)	9 (22.5)	16 (21.6)	4 (18.2)	14 (25.5)
Mutant	26 (13.6)	7 (17.5)	8 (10.8)	3 (13.6)	8 (14.5)
Unknown	122 (63.9)	24 (60.0)	50 (67.6)	15 (68.2)	33 (60.0)
ECOG PS, *n* (%)^c^					
0	82 (42.9)	16 (40.0)	37 (50.0)	7 (31.8)	22 (40.0)
1	62 (32.5)	9 (22.5)	16 (21.6)	13 (59.1)	24 (43.6)
2	2 (1.0)	0	1 (1.4)	1 (4.5)	0
Not documented	45 (23.6)	15 (37.5)	20 (27.0)	1 (4.5)	9 (16.4)
CCI, *n* (%)					
0	137 (71.7)	24 (60.0)	61 (82.4)	17 (77.3)	35 (63.6)
1	38 (19.9)	11 (27.5)	9 (12.2)	5 (22.7)	13 (23.6)
2	11 (5.8)	4 (10.0)	2 (2.7)	0	5 (9.1)
3+	5 (2.6)	1 (2.5)	2 (2.7)	0	2 (3.6)

Abbreviations: CCI, Charlson Comorbidity Index; ECOG PS, Eastern Cooperative Oncology Group performance score; LDH, lactate dehydrogenase; ULN, upper limit of normal.

^a^ Unless otherwise indicated (i.e., median age or age group at index and stage at diagnosis), all demographic and clinical characteristics are those documented on the closest date to the drug index date within a ± 30‐day window.

^b^ Categories for LDH group are not mutually exclusive.

^c^ Nominal *P* = 0.0019 for trend across ECOG PS categories; *P* values for all other listed variables were nonsignificant (*P* > 0.05).

### 3.3 Duration of therapy

Median duration of therapy (95% confidence interval [CI]) for the unadjusted full population (*N* = 191) was 10.3 (9.4–10.7) months, with a relatively consistent range across the cohorts from a low of 8.3 (5.2–10.6) months in cohort 3 to a high of 10.7 (9.8–11.3) months in cohort 4 (Table 2). Consistent with the unadjusted full population, no noteworthy variances in treatment duration were observed in the matched C1:C4 and C2:C4 analyses (Table S1).

**TABLE 2:** Duration of therapy for the unadjusted full population (*N* = 191)

	All patients (*N* = 191)	Cohort 1 (*N* = 40)	Cohort 2 (*N* = 74)	Cohort 3 (*N* = 22)	Cohort 4 (*N* = 55)
Events, *n* (%)^a^	168 (88.0)	37 (92.5)	67 (90.5)	17 (77.3)	47 (85.5)
Median (95% CI), months	10.3 (9.4–10.7)	10.2 (5.3–10.8)	10.0 (7.6–11.0)	8.3 (5.2–10.6)	10.7 (9.8–11.3)

Abbreviation: CI, confidence interval.

^a^ Events were defined as patients switching to another systemic therapy or stopping treatment without reinitiating treatment. Censoring was performed if treatment was continued through the study end date.

### 3.4 Safety

Overall, 113 patients (59.2%) in the unadjusted full population experienced ≥1 TRAE within 12 months of initiation of adjuvant nivolumab; 35 patients (18.3%) experienced ≥1 ‘significant’ TRAE (Table 3). The most common TRAE was fatigue (29.8%) and the most common ‘significant’ TRAE was diarrhea (5.8%). Across the 4 cohorts, proportions of patients experiencing a TRAE were relatively consistent, (range, 54.5%–60.1%) as were the most frequent events (fatigue, rash, diarrhea, hypothyroidism, nausea, and pruritus). However, proportions experiencing ‘significant’ TRAEs showed a variance across the cohorts (Table 3), related primarily to a lower incidence of ‘significant’ diarrhea in cohorts 1 and 2. These safety findings were supported by data for the matched C1:C4 and C2:C4 patient populations (Table S2).

**TABLE 3:** Summary of TRAEs and ‘Significant’ TRAEs experienced within 12 months of initiation of adjuvant nivolumab in the unadjusted full population (*N* = 191)

	All patients (*N* = 191)	Cohort 1 (*N* = 40)	Cohort 2 (*N* = 74)	Cohort 3 (*N* = 22)	Cohort 4 (*N* = 55)
At least 1 TRAE^a^, *n* (%)	113 (59.2)	24 (60.0)	45 (60.1)	12 (54.5)	32 (58.2)
Fatigue	57 (29.8)	14 (35.0)	19 (25.7)	5 (22.7)	19 (34.5)
Rash	31 (16.2)	7 (17.5)	14 (18.9)	3 (13.6)	7 (12.7)
Diarrhea	30 (15.7)	4 (10.0)	13 (17.6)	4 (18.2)	9 (16.4)
Hypothyroidism	20 (10.5)	7 (17.5)	4 (5.4)	2 (9.1)	7 (12.7)
Nausea	15 (7.9)	2 (5.0)	4 (5.4)	3 (13.6)	6 (10.9)
Pruritus	12 (6.3)	2 (5.0)	6 (8.1)	1 (4.5)	3 (5.5)
Abdominal pain	6 (3.1)	0	2 (2.7)	1 (4.5)	3 (5.5)
Colitis	5 (2.6)	2 (5.0)	2 (2.7)	0	1 (1.8)
Headache	5 (2.6)	0	2 (2.7)	1 (4.5)	2 (3.6)
Thyroiditis	5 (2.6)	0	1 (1.4)	1 (4.5)	3 (5.5)
Hypersensitivity (infusion reaction)	4 (2.1)	0	1 (1.4)	1 (4.5)	2 (3.6)
Hyperthyroidism	3 (1.6)	0	1 (1.4)	0	2 (3.6)
Increased AST	3 (1.6)	1 (2.5)	0	0	2 (3.6)
Diabetes	2 (1.0)	1 (2.5)	1 (1.4)	0	0
Increased ALT	2 (1.0)	0	0	0	2 (3.6)
Adrenal insufficiency	1 (0.5)	0	0	0	1 (1.8)
Pancreatitis	1 (0.5)	0	1 (1.4)	0	0
Pneumonitis	1 (0.5)	1 (2.5)	0	0	0
Fever	1 (0.5)	0	1 (1.4)	0	0
Renal failure	1 (0.5)	0	0	1 (4.5)	0
Other	53 (27.7)	7 (17.5)	20 (27.0)	9 (40.9)	17 (30.9)
At least 1 ‘significant’ TRAE,^b^ *n* (%)	35 (18.3)	7 (17.5)	11 (14.9)	6 (27.3)	11 (20.0)
Diarrhea	11 (5.8)	0	3 (4.1)	2 (9.1)	6 (10.9)
Fatigue	8 (4.2)	1 (2.5)	2 (2.7)	1 (4.5)	4 (7.3)
Abdominal pain	6 (3.1)	0	2 (2.7)	1 (4.5)	3 (5.5)
Nausea	6 (3.1)	1 (2.5)	1 (1.4)	1 (4.5)	3 (5.5)
Rash	5 (2.6)	0	4 (5.4)	1 (4.5)	0
Hypothyroidism	5 (2.6)	1 (2.5)	0	1 (4.5)	3 (5.5)
Colitis	3 (1.6)	1 (2.5)	1 (1.4)	0	1 (1.8)
Increased AST	2 (1.0)	1 (2.5)	0	0	1 (1.8)
Pruritus	2 (1.0)	0	2 (2.7)	0	0
Hypersensitivity (infusion reaction)	2 (1.0)	0	1 (1.4)	0	1 (1.8)
Increased ALT	1 (0.5)	0	0	0	1 (1.8)
Diabetes	1 (0.5)	1 (2.5)	0	0	0
Headache	1 (0.5)	0	1 (1.4)	0	0
Pneumonitis	1 (0.5)	1 (2.5)	0	0	0
Renal failure	1 (0.5)	0	0	1 (4.5)	0
Other	16 (8.4)	2 (5.0)	4 (5.4)	6 (27.3)	4 (7.3)

Abbreviations: ALT, alanine aminotransferase; AST, aspartate aminotransferase; TRAE, treatment‐related adverse event.

^a^ Protocol defined as any AE that occurred after the initiation of adjuvant nivolumab for melanoma and that was explicitly attributed to adjuvant nivolumab as documented in a patient's medical chart. The listed individual TRAEs are based on TRAEs reported for adjuvant nivolumab in CheckMate 238^1^; any other TRAEs were included in the Other category.

^b^ Protocol defined as any TRAE (as defined above) that led to ≥1 of the following outcomes: withheld dose, altered dose and/or schedule, permanent treatment discontinuation, hospitalization, or emergency department visit. The listed individual ‘significant’ TRAEs are based on TRAEs reported for adjuvant nivolumab in CheckMate 238^1^;any other ‘significant’ TRAEs were included in the Other category.

### 3.5 Reasons for treatment discontinuation

In total, 70 of the 191 patients (36.6%) in the unadjusted full population prematurely discontinued their adjuvant nivolumab dosing regimen during the study period, most commonly due to disease progression (11.5%), toxicity (9.9%), patient and/or physician preference (5.8%), or for undocumented reasons (5.2%; Table 4). A small number of patients also discontinued due to financial/insurance related reasons, a decline in performance, or death. Overall proportions of patients who prematurely discontinued adjuvant nivolumab were relatively consistent across the 4 cohorts. However, there were variances between some cohorts in the proportions discontinuing due to disease progression, toxicity, and patient and/or physician preference (Table 4). Similar patterns of discontinuation and between‐cohort variances were observed in the matched C1:C4 and C2:C4 analyses (Table S3).

**TABLE 4:** Reasons for treatment discontinuation in unadjusted full population (*N* = 191)

	All patients (*N* = 191)	Cohort 1 (*N* = 40)	Cohort 2 (*N* = 74)	Cohort 3 (*N* = 22)	Cohort 4 (*N* = 55)
Treatment is ongoing, *n* (%)	15 (7.9)	2 (5.0)	4 (5.4)	5 (22.7)	4 (7.3)
Completed planned treatment, *n* (%)	106 (55.5)	25 (62.5)	45 (60.8)	8 (36.4)	28 (50.9)
Prematurely discontinued,^a^ *n* (%)	70 (36.6)	13 (32.5)	25 (33.8)	9 (40.9)	23 (41.8)
Progressive disease	22 (11.5)	4 (10.0)	2 (2.7)	3 (13.6)	13 (23.6)
Toxicity	19 (9.9)	5 (12.5)	8 (10.8)	2 (9.1)	4 (7.3)
Not documented	10 (5.2)	2 (5.0)	4 (5.4)	0	4 (7.3)
Patient preference	7 (3.7)	0	4 (5.4)	2 (9.1)	1 (1.8)
Other	5 (2.6)	0	2 (2.7)	2 (9.1)	1 (1.8)
Physician preference	4 (2.1)	0	4 (5.4)	0	0
Financial/insurance related	3 (1.6)	2 (5.0)	1 (1.4)	0	0
Death	1 (0.5)	0	0	0	1 (1.8)
Decline in performance	1 (0.5)	0	1 (1.4)	0	0

^a^ Patients could have >1 reason for discontinuation recorded.

### 3.6 Sensitivity analyses

Patient demographic and clinical characteristics in the sensitivity full population mirrored those of the unadjusted full population, including the observed ECOG PS variance resulting from an imbalance of patients with missing ECOG data (data not shown); similar to the unadjusted populations, this variance was notably reduced in the sensitivity‐matched C2:C4 population. Duration of therapy, safety and reasons for treatment discontinuation outcomes from the sensitivity analyses are shown in Tables S4–S9. In general, the findings from the unadjusted full population were supported by those from the various sensitivity population analyses. One exception was that the duration of therapy was noticeably shorter in sensitivity cohort 2, particularly compared with cohorts 1 and 4 (see Tables S4 and S5). In the sensitivity full analysis, median treatment duration (95% CI) was 6.7 (5.3–7.6) months for cohort 2 versus 10.2 (5.3–10.8) and 10.7 (9.8–11.3) months for cohorts 1 and 4, respectively; in the sensitivity‐matched C2:C4 population analysis, median treatment durations (95% CI) were 7.2 (5.0–7.8) and 10.7 (9.8–11.3) months, respectively.

## 4 DISCUSSION

This study is among the first to provide real‐world data on the utilization and safety of flat‐dose nivolumab and is the first to specifically examine outcomes with adjuvant nivolumab 480 mg Q4W for melanoma in the US community oncology setting. Overall, regardless of the population of interest (unadjusted, propensity matched, or sensitivity), outcomes from this analysis suggest that the 2 flat‐dose regimens (240 mg Q2W and 480 mg Q4W) are comparable to each other and to the weight‐based dosing regimen (3 mg/kg Q2W) in terms of real‐world duration of therapy, safety, and reasons for treatment discontinuation.

Baseline characteristics were largely similar across the 4 nivolumab dosing cohorts, irrespective of the population of interest. A notable exception was ECOG PS, which showed a statistically significant variance across the cohorts, driven mostly by an imbalance in the proportions of patients with missing ECOG PS data. Data on performance status are often not routinely collected in the community oncology setting and missing data are, as a result, common in real‐world databases. Importantly, this variance was reduced (and not statistically significant) in all of the full or sensitivity‐matched analyses (data not shown).

In the unadjusted full and matched population analyses, the duration of therapy was relatively consistent across the cohorts, ranging between approximately 8 and 11 months. However, in the relevant sensitivity analyses (i.e., conducted after excluding the 22 patients from cohort 2 who switched to nivolumab 480 mg Q4W after the study's predefined enrollment period), median treatment duration was shorter for the remaining patients in cohort 2. This is as expected given that the patients who switched to nivolumab 480 mg Q4W during the predefined enrollment period had already received adjuvant nivolumab for a period of time when enrolled into the study.

Safety of the different adjuvant nivolumab regimens evaluated in this study was relatively consistent in terms of the overall incidence of TRAEs, an observation that supports previous studies that showed similar predicted safety profiles for the 2 flat‐dose regimens (240 mg Q2W and 480 mg Q4W) and the weight‐based dosing regimen (3 mg/kg Q2W).^3^, ^9^ No new safety signals were identified in this study, with the most commonly reported treatment‐related events across the 4 cohorts being relatively consistent with those reported for the 3 mg/kg dose in both the CheckMate 238 trial of adjuvant nivolumab and a pooled safety analysis of nivolumab for advanced melanoma.^1^, ^15^ Based on currently available data, these events also appear to be generally aligned with those reported for adjuvant nivolumab 480 Q4W in the CheckMate 915 trial of patients with resected stage IIIB‐D/IV melanoma.^12^, ^13^

Despite small patient numbers in certain populations, there was a consistently lower observed incidence of ‘significant’ diarrhea in patients receiving the nivolumab 480 mg Q4W flat dose (cohorts 1 and 2) compared with those receiving nivolumab 3 mg/kg Q2W (cohort 3) or nivolumab 240 mg Q2W (cohort 4). As the 480 mg flat dose was the most recent adjuvant regimen to be approved, this may reflect greater physician experience with managing immune‐mediated gastrointestinal AEs, such that the diarrhea was managed before becoming ‘significant.’ Also, the lower incidence of ‘significant’ diarrhea among patients in cohort 2 is not unexpected. Temporal analyses from a pooled safety analysis of nivolumab for advanced melanoma found that the vast majority of new gastrointestinal events occurred within the first 4 months of treatment,^15^ suggesting that at least some of the patients enrolled into cohort 2 might have been at reduced risk of new gastrointestinal events given the length of time they had already been receiving nivolumab. Indeed, for the same reason, it is perhaps surprising that the incidence of TRAEs was not consistently lower in cohort 2. In another previous study of the safety of different nivolumab dosing regimens, the overall incidence of TRAEs and treatment‐related serious AEs was higher in patients receiving de novo 3 mg/kg Q2W in several clinical trials than reported for a cohort of these patients after subsequent transition to a 480 mg Q4W regimen.^4^ One reason for not seeing a lower overall incidence of TRAEs in cohort 2 may be because even in the sensitivity cohort 2 population, there remained 32 patients with a drug index date during their prior treatment with nivolumab 240 mg or 3 mg/kg Q2W. As such, at least some of the safety data for these patients would have been related to those initial doses rather than the subsequent 480 mg Q4W regimen. It is important to note that any differences between the cohorts in rates of individual treatment‐related events should be interpreted with caution, given the small sample sizes. Additionally, because information on CTCAE grading of TRAEs was not available in the source database, the ability to further examine safety profiles across the cohorts was limited.

Across the cohorts and in all of the conducted analyses, the most common reasons for discontinuation were disease progression and study drug toxicity, consistent with findings from the CheckMate 238 trial.^1^ Although random fluctuations would be expected with the relatively small population sizes, there appeared to be a consistently lower proportion of patients in cohort 2 who discontinued because of disease progression. This likely reflects the fact that these patients had already been receiving adjuvant nivolumab before switching to the nivolumab 480 mg Q4W flat dose, and any patients susceptible to rapid progression on nivolumab would not have entered the current study (as they would have progressed before enrollment).

All of the reported study outcomes should be considered in light of the small sample sizes for individual cohorts, which were further decreased in the propensity matched and sensitivity analyses; the retrospective design of the study, which inherently reduces the level of evidence versus a prospective study; and the fact that the study was not designed to examine switching between dosing regimens. It is also important to acknowledge that propensity matched comparisons could not be conducted between the flat‐dose and weight‐based dosing cohorts due to limited patient numbers in cohort 3, thereby reducing the robustness of any direct comparison of flat versus weight‐based dosing in this study. Furthermore, it should be noted that the sensitivity cohort 2 population included 32 patients with a drug index date during their prior treatment with nivolumab 240 mg or 3 mg/kg Q2W; as such, outcomes for this population would have been influenced in part by those prior dose regimens. Additionally, outcomes could have been influenced by both provider treatment bias (e.g., a physician administering the 480 mg Q4W flat dose to patients with specific characteristics vs the 3 mg/kg Q2W regimen) and attribution bias (e.g., a physician being more likely to attribute an AE to adjuvant nivolumab for patients receiving the 480 mg Q4W regimen than other regimens due to the higher dose). Indeed, the observed lower mean body weight for patients in cohort 3 may suggest an element of provider treatment bias whereby lighter patients were deemed more suitable for weight‐based dosing. Finally, while the propensity score matching process was designed to reduce the potential for selection bias and balance the population, there remains the possibility that the outcomes were influenced either by other collected characteristics not used for the propensity score matching or by characteristics not available in the US Oncology Network iKM database or patient charts.

In summary, despite these limitations, our study findings suggest that the 2 flat‐dose nivolumab regimens (240 mg Q2W and 480 mg Q4W) have real‐world safety and tolerability profiles that are comparable to each other and to the well‐documented profile for the weight‐based dosing regimen (3 mg/kg Q2W), adding to the existing data supporting their approval and use in the adjuvant setting for melanoma.

## AUTHOR CONTRIBUTIONS

WS: Conceptualization, data curation, investigation, methodology, project administration, supervision, validation, writing—original draft, and writing—review and editing. NJR: Conceptualization, investigation, writing—original draft, and writing—review and editing. LC: Conceptualization, formal analysis, investigation, writing—original draft, and writing—review and editing. BS: Conceptualization, methodology, writing—original draft, and writing—review and editing. CD: Conceptualization, writing—original draft, and writing—review and editing. AM: Conceptualization, writing—original draft, and writing—review and editing. SK: Conceptualization, writing—original draft, and writing—review and editing. TP: Conceptualization, writing—original draft, and writing—review and editing. JSW: Conceptualization, validation, writing—original draft, and writing—review and editing.

## FUNDING INFORMATION

This study was supported by Bristol Myers Squibb (Princeton, NJ) and Ono Pharmaceutical Company Ltd (Osaka, Japan).

## CONFLICT OF INTEREST

WS: data safety monitoring boards for Regeneron, Sanofi Genzyme, Immunicum; advisory boards for Novartis and Eisai; and speakers bureaus for Bristol Myers Squibb (BMS), Merck, Sanofi Aventis, Regeneron, and Novartis. NJR and LC: employment by McKesson Life Sciences. BS, CD, AM, SK, and TP: employment by BMS. JSW: consultancy for Merck, Genentech, AstraZeneca, GSK, Novartis, Nektar, Celldex, Incyte, Biond, ImCheck, Sellas, Evaxion, and EMD Serono; advisory boards for BMS, CytomX, Incyte, ImCheck, Biond, Sellas, Instil Bio, OncoC4, and NexImmune; and equity in Biond, Evaxion, Instil Bio, OncoC4, and NexImmune.

### ETHICS APPROVAL

This retrospective observational research study was conducted in accordance with relevant legal and regulatory requirements, to ensure the protection of the rights and privacy of the patients whose records were included in the study. The protocol received appropriate exemption and waiver of informed consent from the centralized US Oncology Institutional Review Board.

## Supporting information


Table S1‐S9

## Data Availability

Bristol Myers Squibb’s policy on data sharing may be found at https://www.bms.com/researchers‐and‐partners/independent‐research/data‐sharing‐request‐process.html.
