# Supplementary material for: Real‐World nivolumab dosing patterns and safety outcomes in patients receiving adjuvant therapy for melanoma
Source: Cancer Med. 2022 Jul 25;12(3):2378–88. doi: 10.1002/cam4.5061 (PMC9939122; doi:10.1002/cam4.5061)
Supplement: Supplementary file 1 — Table S1‐S9 [file CAM4-12-2378-s001.docx]

**SUPPORTING INFORMATION** (ONLINE ONLY)

**Real-World Nivolumab Dosing Patterns and Safety Outcomes in Patients Receiving Adjuvant Therapy for Melanoma**

Samlowski W, Robert NJ, Chen L, et al.

**CONTENTS**

[**Supporting Table 1.** Duration of Therapy for Patients in the Matched C1:C4 and C2:C4 Populations 2](#_Toc93939278)

[**Supporting Table 2.** Summary of TRAEs and ‘Significant’ TRAEs Experienced Within 12 Months of Initiation of Adjuvant Nivolumab Among Patients in the Matched C1:C4 and C2:C4 Populations 3](#_Toc93939279)

[**Supporting Table 3.** Reasons for Treatment Discontinuation Among Patients in the Matched C1:C4 and C2:C4 Populations 4](#_Toc93939280)

[**Supporting Table 4.** Duration of Therapy for the Sensitivity Full Population^a^ 5](#_Toc93939281)

[**Supporting Table 5.** Duration of Therapy for the Sensitivity-Matched C2:C4 Population 6](#_Toc93939282)

[**Supporting Table 6.** Summary of TRAEs and ‘Significant’ TRAEs Experienced Within 12 Months of Initiation of Adjuvant Nivolumab Among Patients in the Sensitivity full population^a^ 7](#_Toc93939283)

[**Supporting Table 7.** Summary of TRAEs and ‘Significant’ TRAEs Experienced Within 12 Months of Initiation of Adjuvant Nivolumab Among Patients in the Sensitivity-Matched C2:C4 Population 9](#_Toc93939284)

[**Supporting Table 8.** Reasons for Treatment Discontinuation in the Sensitivity Full Population^a^ 10](#_Toc93939285)

[**Supporting Table 9.** Reasons for Treatment Discontinuation in the Sensitivity-Matched C2:C4 Population 11](#_Toc93939286)

# **Supporting Table 1.** Duration of Therapy for Patients in the Matched C1:C4 and C2:C4 Populations

|  | Cohort 1 (N = 37) | Cohort 4  (N = 37) | Cohort 2  (N = 53) | Cohort 4 (N = 53) |
| --- | --- | --- | --- | --- |
| Events, n (%)^a^ | 36 (97.3) | 31 (83.8) | 49 (92.5) | 45 (84.9) |
| Median (95% CI), months | 10.2 (5.3-10.8) | 10.1 (5.2-11.1) | 9.5 (7.4-11.0) | 10.7 (9.8-11.3) |

Abbreviation: CI, confidence interval.

^a^Events were defined as patients switching to another systemic therapy or stopping treatment without reinitiating treatment. Censoring was performed if treatment was continued through the study end date.

# **Supporting Table 2.** Summary of TRAEs and ‘Significant’ TRAEs Experienced Within 12 Months of Initiation of Adjuvant Nivolumab Among Patients in the Matched C1:C4 and C2:C4 Populations

|  | Cohort 1 (N = 37) | Cohort 4  (N = 37) | Cohort 2  (N = 53) | Cohort 4 (N = 53) |
| --- | --- | --- | --- | --- |
| At least 1 TRAE,^a^ n (%) | 24 (64.9) | 21 (56.8) | 32 (60.4) | 31 (58.5) |
| Fatigue | 14 (37.8) | 13 (35.1) | 13 (24.5) | 19 (35.8) |
| Rash | 7 (18.9) | 5 (13.5) | 10 (18.9) | 6 (11.3) |
| Diarrhea | 4 (10.8) | 7 (18.9) | 8 (15.1) | 9 (17.0) |
| Hypothyroidism | 7 (18.9) | 4 (10.8) | 3 (5.7) | 7 (13.2) |
| Nausea | 2 (5.4) | 3 (8.1) | 4 (7.5) | 6 (11.3) |
| Pruritus | 2 (5.4) | 3 (8.1) | 5 (9.4) | 3 (5.7) |
| Abdominal pain | 0 | 3 (8.1) | 2 (3.8) | 3 (5.7) |
| Colitis | 2 (5.4) | 1 (2.7) | 1 (1.9) | 1 (1.9) |
| Hypersensitivity (infusion reaction) | 0 | 2 (5.4) | 1 (1.9) | 2 (3.8) |
| Increased AST | 1 (2.7) | 1 (2.7) | 0 | 2 (3.8) |
| Adrenal insufficiency | 0 | 1 (2.7) | 0 | 1 (1.9) |
| Headache | 0 | 1 (2.7) | 0 | 2 (3.8) |
| Hyperthyroidism | 0 | 1 (2.7) | 1 (1.9) | 2 (3.8) |
| Increased ALT | 0 | 1 (2.7) | 0 | 2 (3.8) |
| Thyroiditis | 0 | 1 (2.7) | 1 (1.9) | 3 (5.7) |
| Diabetes | 1 (2.7) | 0 | 1 (1.9) | 0 |
| Pneumonitis | 1 (2.7) | 0 | 0 | 0 |
| Pancreatitis | 0 | 0 | 1 (1.9) | 0 |
| Other | 7 (18.9) | 9 (24.3) | 14 (26.4) | 16 (30.2) |
| At least 1 ‘significant’ TRAE,^b^ n (%) | 7 (18.9) | 7 (18.9) | 10 (18.9) | 11 (20.8) |
| Diarrhea | 0 | 4 (10.8) | 2 (3.8) | 6 (11.3) |
| Fatigue | 1 (2.7) | 3 (8.1) | 2 (3.8) | 4 (7.5) |
| Nausea | 1 (2.7) | 3 (8.1) | 1 (1.9) | 3 (5.7) |
| Abdominal pain | 0 | 3 (8.1) | 2 (3.8) | 3 (5.7) |
| Hypothyroidism | 1 (2.7) | 1 (2.7) | 0 | 3 (5.7) |
| Rash | 0 | 0 | 3 (5.7) | 0 |
| Colitis | 1 (2.7) | 1 (2.7) | 1 (1.9) | 1 (1.9) |
| Increased AST | 1 (2.7) | 1 (2.7) | 0 | 1 (1.9) |
| Increased ALT | 0 | 1 (2.7) | 0 | 1 (1.9) |
| Hypersensitivity (infusion reaction) | 0 | 1 (2.7) | 1 (1.9) | 1 (1.9) |
| Diabetes | 1 (2.7) | 0 | 0 | 0 |
| Pneumonitis | 1 (2.7) | 0 | 0 | 0 |
| Pruritus | 0 | 0 | 1 (1.9) | 0 |
| Other | 2 (5.4) | 2 (5.4) | 4 (7.5) | 4 (7.5) |

Abbreviations: ALT, alanine aminotransferase; AST, aspartate aminotransferase; TRAE, treatment-resistant adverse event.

^a^Protocol-defined as any adverse event that occurred after the initiation of adjuvant nivolumab for melanoma and that was explicitly attributed to adjuvant nivolumab as documented in a patient’s medical chart. The listed individual TRAEs are based on TRAEs reported for adjuvant nivolumab in CheckMate 238; any other TRAEs were included in the Other category.

^b^Protocol-defined as any TRAE that led to ≥1 of the following outcomes: withheld dose, altered dose and/or schedule, permanent treatment discontinuation, hospitalization, or emergency department visit. The listed individual ‘significant’ TRAEs are based on TRAEs reported for adjuvant nivolumab in CheckMate 238; any other ‘significant’ TRAEs were included in the Other category.

# **Supporting Table 3.** Reasons for Treatment Discontinuation Among Patients in the Matched C1:C4 and C2:C4 Populations

|  | Cohort 1 (N = 37) | Cohort 4  (N = 37) | Cohort 2  (N = 53) | Cohort 4 (N = 53) |
| --- | --- | --- | --- | --- |
| Treatment is ongoing, n (%) | 2 (5.4) | 3 (8.1) | 3 (5.7) | 4 (7.5) |
| Completed planned treatment, n (%) | 24 (64.9) | 16 (43.2) | 29 (54.7) | 27 (50.9) |
| Prematurely discontinued,^a^ n (%) | 11 (29.7) | 18 (48.6) | 21 (39.6) | 22 (41.5) |
| Progressive disease | 4 (10.8) | 11 (29.7) | 2 (3.8) | 12 (22.6) |
| Toxicity | 5 (13.5) | 3 (8.1) | 8 (15.1) | 4 (7.5) |
| Not documented | 0 | 3 (8.1) | 2 (3.8) | 4 (7.5) |
| Patient preference | 0 | 1 (2.7) | 3 (5.7) | 1 (1.9) |
| Other | 0 | 0 | 1 (1.9) | 1 (1.9) |
| Physician preference | 0 | 0 | 4 (7.5) | 0 |
| Financial/insurance related | 2 (5.4) | 0 | 1 (1.9) | 0 |
| Death | 0 | 1 (2.7) | 0 | 1 (1.9) |
| Decline in performance | 0 | 0 | 1 (1.9) | 0 |

^a^Patients could have >1 reason for discontinuation recorded.

# **Supporting Table 4.** Duration of Therapy for the Sensitivity Full Population^a^

|  | Cohort 1 (N = 40) | Cohort 2  (N = 52) | Cohort 3  (N = 22) | Cohort 4 (N = 55) |
| --- | --- | --- | --- | --- |
| Events, n (%)^b^ | 37 (92.5) | 47 (90.4) | 17 (77.3) | 47 (85.5) |
| Median (95% CI), months | 10.2 (5.3-10.8) | 6.7 (5.3-7.6) | 8.3 (5.2-10.6) | 10.7 (9.8-11.3) |

Abbreviation: CI, confidence interval.

^a^All data with exception of sensitivity cohort 2 are identical to those shown in Table 2 in main article. Sensitivity cohort 2 excludes 22 patients from the full unadjusted cohort 2 who deviated from the planned study design.

^b^Events were defined as patients switching to another systemic therapy or stopping treatment without reinitiating treatment. Censoring was performed if treatment was continued through the study end date.

# **Supporting Table 5.** Duration of Therapy for the Sensitivity-Matched C2:C4 Population

|  | Cohort 2 (N = 35) | Cohort 4 (N = 35) |
| --- | --- | --- |
| Events, n (%)^a^ | 33 (94.3) | 29 (82.9) |
| Median (95% CI), months | 7.2 (5.0-7.8) | 10.7 (9.8-11.3) |

Abbreviation: CI, confidence interval.

^a^Events were defined as patients switching to another systemic therapy or stopping treatment without reinitiating treatment. Censoring was performed if treatment was continued through the study end date.

# **Supporting Table 6.** Summary of TRAEs and ‘Significant’ TRAEs Experienced Within 12 Months of Initiation of Adjuvant Nivolumab Among Patients in the Sensitivity full population^a^

|  | Cohort 1 (N = 40) | Cohort 2  (N = 52) | Cohort 3  (N = 22) | Cohort 4 (N = 55) |
| --- | --- | --- | --- | --- |
| At least 1 TRAE,^b^ n (%) | 24 (60.0) | 35 (67.3) | 12 (54.5) | 32 (58.2) |
| Fatigue | 14 (35.0) | 17 (32.7) | 5 (22.7) | 19 (34.5) |
| Rash | 7 (17.5) | 11 (21.2) | 3 (13.6) | 7 (12.7) |
| Diarrhea | 4 (10.0) | 9 (17.3) | 4 (18.2) | 9 (16.4) |
| Hypothyroidism | 7 (17.5) | 3 (5.8) | 2 (9.1) | 7 (12.7) |
| Nausea | 2 (5.0) | 3 (5.8) | 3 (13.6) | 6 (10.9) |
| Pruritus | 2 (5.0) | 5 (9.6) | 1 (4.5) | 3 (5.5) |
| Abdominal pain | 0 | 1 (1.9) | 1 (4.5) | 3 (5.5) |
| Colitis | 2 (5.0) | 2 (3.8) | 0 | 1 (1.8) |
| Headache | 0 | 2 (3.8) | 1 (4.5) | 2 (3.6) |
| Thyroiditis | 0 | 1 (1.9) | 1 (4.5) | 3 (5.5) |
| Hypersensitivity (infusion reaction) | 0 | 1 (1.9) | 1 (4.5) | 2 (3.6) |
| Hyperthyroidism | 0 | 1 (1.9) | 0 | 2 (3.6) |
| Increased AST | 1 (2.5) | 0 | 0 | 2 (3.6) |
| Diabetes | 1 (2.5) | 1 (1.9) | 0 | 0 |
| Increased ALT | 0 | 0 | 0 | 2 (3.6) |
| Adrenal insufficiency | 0 | 0 | 0 | 1 (1.8) |
| Pancreatitis | 0 | 1 (1.9) | 0 | 0 |
| Pneumonitis | 1 (2.5) | 0 | 0 | 0 |
| Fever | 0 | 1 (1.9) | 0 | 0 |
| Renal failure | 0 | 0 | 1 (4.5) | 0 |
| Other | 7 (17.5) | 15 (28.8) | 9 (40.9) | 17 (30.9) |
| At least 1 ‘significant’ TRAE,^c^ n (%) | 7 (17.5) | 9 (17.3) | 6 (27.3) | 11 (20.0) |
| Diarrhea | 0 | 2 (3.8) | 2 (9.1) | 6 (10.9) |
| Fatigue | 1 (2.5) | 2 (3.8) | 1 (4.5) | 4 (7.3) |
| Abdominal pain | 0 | 1 (1.9) | 1 (4.5) | 3 (5.5) |
| Nausea | 1 (2.5) | 1 (1.9) | 1 (4.5) | 3 (5.5) |
| Rash | 0 | 4 (7.7) | 1 (4.5) | 0 |
| Hypothyroidism | 1 (2.5) | 0 | 1 (4.5) | 3 (5.5) |
| Colitis | 1 (2.5) | 1 (1.9) | 0 | 1 (1.8) |
| Increased AST | 1 (2.5) | 0 | 0 | 1 (1.8) |
| Pruritus | 0 | 2 (3.8) | 0 | 0 |
| Hypersensitivity (infusion reaction) | 0 | 1 (1.9) | 0 | 1 (1.8) |
| Increased ALT | 0 | 0 | 0 | 1 (1.8) |
| Diabetes | 1 (2.5) | 0 | 0 | 0 |
| Headache | 0 | 1 (1.9) | 0 | 0 |
| Pneumonitis | 1 (2.5) | 0 | 0 | 0 |
| Renal failure | 0 | 0 | 1 (4.5) | 0 |
| Other | 2 (5.0) | 3 (5.8) | 6 (27.3) | 4 (7.3) |

Abbreviations: ALT, alanine aminotransferase; AST, aspartate aminotransferase; TRAE, treatment-related adverse event.

^a^All data with exception of sensitivity cohort 2 are identical to those shown in Table 3 in main article. Sensitivity cohort 2 excludes 22 patients from the full unadjusted cohort 2 who deviated from the planned study design.

^b^Protocol-defined as any adverse event that occurred after the initiation of adjuvant nivolumab for melanoma and that was explicitly attributed to adjuvant nivolumab as documented in a patient’s medical chart. The listed individual TRAEs are based on TRAEs reported for adjuvant nivolumab in CheckMate 238; any other TRAEs were included in the Other category.

^c^Protocol-defined as any TRAE that led to ≥1 of the following outcomes: withheld dose, altered dose and/or schedule, permanent treatment discontinuation, hospitalization, or emergency department visit. The listed individual ‘significant’ TRAEs are based on TRAEs reported for adjuvant nivolumab in CheckMate 238; any other ‘significant’ TRAEs were included in the Other category.

# **Supporting Table 7.** Summary of TRAEs and ‘Significant’ TRAEs Experienced Within 12 Months of Initiation of Adjuvant Nivolumab Among Patients in the Sensitivity-Matched C2:C4 Population

|  | Cohort 2  (N = 35) | Cohort 4 (N = 35) |
| --- | --- | --- |
| At least 1 TRAE,^a^ n (%) | 23 (65.7) | 19 (54.3) |
| Fatigue | 11 (31.4) | 9 (25.7) |
| Rash | 8 (22.9) | 4 (11.4) |
| Diarrhea | 4 (11.4) | 7 (20.0) |
| Hypothyroidism | 2 (5.7) | 4 (11.4) |
| Nausea | 2 (5.7) | 4 (11.4) |
| Pruritus | 4 (11.4) | 2 (5.7) |
| Thyroiditis | 1 (2.9) | 3 (8.6) |
| Headache | 1 (2.9) | 2 (5.7) |
| Hypersensitivity (infusion reaction) | 1 (2.9) | 2 (5.7) |
| Abdominal pain | 0 | 2 (5.7) |
| Colitis | 1 (2.9) | 1 (2.9) |
| Hyperthyroidism | 1 (2.9) | 1 (2.9) |
| Increased ALT | 0 | 2 (5.7) |
| Increased AST | 0 | 2 (5.7) |
| Diabetes | 1 (2.9) | 0 |
| Pancreatitis | 1 (2.9) | 0 |
| Other | 9 (25.7) | 11 (31.4) |
| At least 1 ‘significant’ TRAE,^b^ n (%) | 7 (20.0) | 8 (22.9) |
| Diarrhea | 1 (2.9) | 5 (14.3) |
| Fatigue | 2 (5.7) | 2 (5.7) |
| Rash | 3 (8.6) | 0 |
| Hypothyroidism | 0 | 3 (8.6) |
| Abdominal pain | 0 | 2 (5.7) |
| Colitis | 1 (2.9) | 1 (2.9) |
| Nausea | 1 (2.9) | 1 (2.9) |
| Hypersensitivity (infusion reaction) | 1 (2.9) | 1 (2.9) |
| Increased AST | 0 | 1 (2.9) |
| Pruritus | 1 (2.9) | 0 |
| Increased ALT | 0 | 1 (2.9) |
| Other | 3 (8.6) | 3 (8.6) |

Abbreviations: ALT, alanine aminotransferase; AST, aspartate aminotransferase; TRAE, treatment-related adverse event.

^a^Protocol-defined as any adverse event that occurred after the initiation of adjuvant nivolumab for melanoma and that was explicitly attributed to adjuvant nivolumab as documented in a patient’s medical chart. The listed individual TRAEs are based on TRAEs reported for adjuvant nivolumab in CheckMate 238; any other TRAEs were included in the Other category.

^b^Protocol-defined as any TRAE that led to ≥1 of the following outcomes: withheld dose, altered dose and/or schedule, permanent treatment discontinuation, hospitalization, or emergency department visit. The listed individual ‘significant’ TRAEs are based on TRAEs reported for adjuvant nivolumab in CheckMate 238; any other ‘significant’ TRAEs were included in the Other category.

# **Supporting Table 8.** Reasons for Treatment Discontinuation in the Sensitivity Full Population^a^

|  | Cohort 1 (N = 40) | Cohort 2  (N = 52) | Cohort 3  (N = 22) | Cohort 4 (N = 55) |
| --- | --- | --- | --- | --- |
| Treatment is ongoing n (%), | 2 (5.0) | 4 (7.7) | 5 (22.7) | 4 (7.3) |
| Completed planned treatment, n (%) | 25 (62.5) | 32 (61.5) | 8 (36.4) | 28 (50.9) |
| Prematurely discontinued,^b^ n (%) | 13 (32.5) | 18 (34.6) | 9 (40.9) | 23 (41.8) |
| Progressive disease | 4 (10.0) | 2 (3.8) | 3 (13.6) | 13 (23.6) |
| Toxicity | 5 (12.5) | 7 (13.5) | 2 (9.1) | 4 (7.3) |
| Not documented | 2 (5.0) | 1 (1.9) | 0 | 4 (7.3) |
| Patient preference | 0 | 3 (5.8) | 2 (9.1) | 1 (1.8) |
| Other | 0 | 1 (1.9) | 2 (9.1) | 1 (1.8) |
| Physician preference | 0 | 1 (1.9) | 0 | 0 |
| Financial/insurance related | 2 (5.0) | 1 (1.9) | 0 | 0 |
| Death | 0 | 0 | 0 | 1 (1.8) |
| Decline in performance | 0 | 1 (1.9) | 0 | 0 |

^a^All data with exception of sensitivity cohort 2 are identical to those shown in Table 4 in main article. Sensitivity cohort 2 excludes 22 patients from the full unadjusted cohort 2 who deviated from the planned study design.

^b^Patients could have >1 reason for discontinuation recorded.

# **Supporting Table 9.** Reasons for Treatment Discontinuation in the Sensitivity-Matched C2:C4 Population

|  | Cohort 2  (N = 35) | Cohort 4 (N = 35) |
| --- | --- | --- |
| Treatment is ongoing, n (%) | 1 (2.9) | 2 (5.7) |
| Completed planned treatment, n (%) | 22 (62.9) | 20 (57.1) |
| Prematurely discontinued,^a^ n (%) | 12 (34.3) | 13 (37.1) |
| Progressive disease | 1 (2.9) | 6 (17.1) |
| Toxicity | 6 (17.1) | 3 (8.6) |
| Not documented | 1 (2.9) | 3 (8.6) |
| Patient preference | 1 (2.9) | 0 |
| Other | 1 (2.9) | 1 (2.9) |
| Physician preference | 1 (2.9) | 0 |
| Financial/insurance related | 1 (2.9) | 0 |
| Decline in performance | 1 (2.9) | 0 |

^a^Patients could have >1 reason for discontinuation recorded.
